# The Neglect of the Poor in Workhouse Infirmaries

**Published:** 1890-08-02

**Authors:** Louisa Twining


### THE NEGLECT OF THE POOR IN WORKHOUSE INFIRMARIES.

By Louisa Twining.

It must seem strange, after the repeated revelations during the past thirty years with regard to the conditions of nursing in our poor-law institutions, that there should be anything left to be said on the subject, or that any complaints and grievances should remain. Yet who can deny that there is still need to urge upon an apathetic or ignorant public a more vigilant watchfulness and earnest interest in these matters? It is to this too prevalent indifference, indeed, that the evils are to be traced, in the present as in the past, and in these days of all-searching inquiry and investigation, it is a remarkable fact that it should be so. When all institutions and managements are on their trial, and are being brought into the light, and under the scrutiny, of publicity, how can we account for the apathy with which the treatment of the most helpless of our sick poor throughout the country is regarded? Even though the interiors of our "State hospitals" may be kept jealously guarded from public inspection, yet there are the published reports of every Board of Guardians to be read in the local papers, and these too often give evidence of a state of things which may well cause us to wonder at the indifference with which they are regarded. And thus we cannot be accused of any breach of confidence in referring to what appears in the public press, supplemented as it is by the statements and experience of our now more than one hundred nurses* scattered through the workhouse infirmaries of the country. Were we to rely upon them alone, we might be accused of one-sidedness, partiality, and the like; but the evidence, uncontradicted, that comes direct from Boards of Guardians, can hardly be denied or gainsaid.

The cause of this indifference is clearly to be traced to the fact that the national mind and conscience (may we not say heart?) has not yet been aroused to the conviction that the workhouse infirmaries or sick wards are really our sole hospitals and refuges for the destitute poor throughout the country; in great measure for even temporary sickness and accident in places far removed from voluntary hospitals; but entirely so for all lasting cases of incurable disease and infirmity; and surely we are justified in calling these the most pitiable and lamentable of all the various forms of human suffering.

Yet, notwithstanding this fact, added to another, that all England is divided into poor law unions, and there is not one of these in which some kindly hearts and sensible minds are not to be found, what is the real state of the case? Go where you will, and begin to speak about the workhouse, with its large proportion of sick and infirm inmates, and how rarely you will find any knowledge of or interest in the subject! Even in the charitable and those who take part in the management of some neighbouring hospital or other institution you will find nothing is known about the poor-law work, unless you come across here and there a stray visitor, who, after many efforts and difficulties, may have been allowed to penetrate into the well-guarded recesses of this "public" institution, supported by the rates, and in which all ought to feel an interest, and acknowledge a claim on their responsibility. It is hardly credible that these same facts should have to be stated and urged at intervals during a period of nearly forty years; but though something has been done, which we acknowledge with thankfulness, we still feel the necessity of repeating the need of what remains to be carried out before we can be satisfied with the existing state of things.

Such repeated and various evidence has been given from time to time of the inefficiency of present arrangements that we should suppose it to be needless to repeat it, did we not see constant proofs of the necessity of further action brought to our notice with almost wearisome reiteration. We must, as so often before, urge the fact that pauper nursing remains at the root of most of the evils and complaints, and probably it is at the present time more completely the cause than at any previous period, because of the vast diminution of able-bodied paupers in our workhouses, a fact at which all must rejoice, but which nevertheless leaves the care of the sick and helpless to those who are themselves incapable from old age, infirmity, or imbecility. Yet it is hardly an exaggeration to say that there is, as a rule, no night nursing of the sick throughout the smaller workhouses of the country, but that of paupers. So inadequate and grudging is even the allowance of salaries for day nurses, who are in many places only now replacing pauper helpers, overlooked by the busy and untrained matron, that it seems hopeless to expect an extension of our plans for night duty, which must be acknowledged to be quite as important as by the day. Let us hear some further evidence on this point.

A lady, fully qualified and trained in hospital management, who visits some of our various nurses at work in country infirmaries, writes thus:—

"There is no night nursing, though there is a very large number of sick to be cared for. If a nurse is called up at night, in cases of urgent necessity, she has to go out of doors

* Workhouse Infirmary Nursing Association.

to fetch the doctor, and to pass between different wards." "There are 68 beds besides midwifery (for one nurse), and the night nursing is done by the paupers."

Let us add a frequent fact, that these said wardsmen and wardswomen do not trouble themselves to get up to the poor helpless creatures under their charge, and leave them to assist themselves, with results that may be better imagined than described, for the day nurse the next morning.

We could fill pages with instances of what we assert, but it can hardly be necessary after all that has already been written. The following case, however, recently given in a daily paper, brings the matter forcibly before us:—A wardswoman of the imbecile and paralytic ward of a northern workhouse has at last been detected by the medical officer in her cruel practices. For some time he had been struck by the frequency of black eyes and frequent contusions among the patients; but on inquiring of the wardswoman he was informed that the injuries he noticed resulted from "tumbling about." At last a severe scalp wound on a paralytic woman brought the truth to light, and the culprit had to confess that it was caused by a blow with a sweeping-brush on the poor old creature's head, because she did not sit down when ordered; it brought her, indeed, to the ground, stunned to insensibility! Yet no one dared to report it, or perhaps was capable of doing so, and chance alone discovered the truth, owing to the position of the wound.

One of our nurses writes of the difficulty of getting a "master" to see and understand the needs in nursing the sick. She had asked for a probationer or assistant, but "he says that I should not do so much of the wardswoman's work, as, for instance, lifting patients in and out of bed, and such like"—(work which every one who understands nursing knows are really *nursing duties*)—"but I really do not see how I am to avoid it when I have such people as I have for deputies." We can hardly wonder that such nurses ask to be allowed to leave their posts and go elsewhere to carry out their profession. Another writes: "I do not see under the present arrangements how it is possible to nurse patients with any satisfaction. If I remained to go on day duty it seems to me that the chief of my work would be to see that the scrubbers did theirs." No wonder that a high and experienced official in poor-law work writes as follows: "Every year I feel how very much more remains to be done, even in London, where there are still some workhouses under the old régime of workhouse management, with pauper nursing not excluded, and therefore fatal to any good system of trained nursing; and in the country, except in a few of the largest towns, almost everything has to be done. Still, the work is spreading, and will spread. The essential thing is to promote, as far as possible, the election of guardians, men or women, who have a higher standard in these things."

Yes, in these last words lies the key to the whole position, for guardians are, or ought to be, the representatives of the electors and the public in every poor-law union, and till they are aroused from their apathy nothing can be hoped for or will be done. Guardians alone have the power to prevent the folly or cruelty of which we have been speaking. They allow, and they can hinder, the appointment of imbeciles for the work of "nursing" the sick, for, under their "care," patients fall out of bed, bones are broken or injuries inflicted, the cause of which often remains undiscovered, while of the untold suffering and misery of which the poor patients cannot or will not speak, who can realise the amount through the long hours of the night?

Even in the decorous and stately leisure of cathedral cities we read of doings and events which it seems incredible the inhabitants can peruse in the daily papers without shame and humiliation at their being brought to light. Incompetent old nurses, who have been muddling over their work for more than twenty years, entrusted not only with the male and female wards, but with midwifery cases also; sometimes deaf, or afflicted in other ways—we need hardly say untrained in the performance of their duties—and when some disgraceful conduct or scandal is brought to light, guardians discuss the question of her dismissal or retention, with or without a pension, the claims of one incompetent and, perhaps, immoral woman outweighing those of poor sufferers who may be reckoned by tens or hundreds!

Not till electors and elected come to realise their duties towards the vast population of sick and helpless beings thrown upon the rates for support in their time of suffering, will there be any remedy found for a state of things that we have become callous to by long continuance and familiarity, but which in any other, or new, branch of work would excite feelings of indignation and shame. Is not the fact of its being national work sufficient to arouse us to a desire that it should be at least decently, if not perfectly, performed?

Is it possible that men of humanity in any rank of life can read such statements as we have given without indignation and horror? And, again, is it credible that so long as thirty-five years ago the real state of things was proclaimed by Mrs. Jameson in her lectures on "Charity" and "the Social Employments of Women," when she said, in describing a large workhouse, "In each ward all the assistance given and all the supervision were in the hands of one nurse and a 'helper,' both chosen from among the pauper women who were supposed to be the least immoral and drunken." And after a further description of the misery she witnessed, she adds: "And these are the sisters of charity to whom our sick poor are confided!" "In one workhouse a poor woman could get no help but by bribery—those who would not pay this tax were neglected, and implored in vain to be turned in their beds." Who will venture to affirm that this is not still too often the case? An article in the *Quarterly Review* of 1855 is quoted by Mrs. Jameson in confirmation of her statements, and so long as the physically, morally, and mentally incapable are set to the sacred task of nursing and tending the sick and the helpless, so long will the past and the present evils remain untouched.

May we add one word as to a remedy that has been already suggested, viz., the appointment of duly qualified *women* as inspectors of the sick wards of county unions? They alone will discover the needs of the sick, and be able to suggest remedies from their own personal experience and insight, for nursing and other deficiencies.

